# Characteristics of and Experience Among People Who Use Take-Home Naloxone in Skåne County, Sweden

**DOI:** 10.3389/fpubh.2022.811001

**Published:** 2022-03-10

**Authors:** Katja Troberg, Pernilla Isendahl, Marianne Alanko Blomé, Disa Dahlman, Anders Håkansson

**Affiliations:** ^1^Division of Psychiatry, Department of Clinical Sciences Lund, Lund University, Lund, Sweden; ^2^Malmö Addiction Centre, Region Skåne, Malmö, Sweden; ^3^Department of Infectious Disease, University Hospital Skåne, Malmö, Sweden; ^4^Regional Office for Communicable Disease Control, Malmö, Sweden; ^5^Center for Primary Health Care Research, Department of Clinical Sciences, Lund University, Region Skåne, Malmö, Sweden

**Keywords:** naloxone, opioids, overdose management, prevention programs, substance use, harm reduction, opioid substitution treatment, needle exchange programs

## Abstract

**Background:**

Opioid overdose related injury or death can be prevented by bystander naloxone administration. For naloxone to be present when and where overdoses occur, opioid prevention education and naloxone distribution (OPEND) must be established on a broad level. This is the 30-month follow-up of the first multi-site naloxone project in Sweden, implemented at 31 sites in the County of Skåne 2018.

**Aim:**

To address participant characteristics and factors associated with returning for naloxone refill and with having used naloxone for overdose reversal. An additional aim was to describe self-reported reasons for naloxone refill and overdose experiences.

**Methods:**

Data were collected during June 2018—December 2020 through questionnaires at baseline and upon naloxone refill of the initial and subsequent naloxone kit. Descriptive statistics was used to address participant characteristics, those returning for naloxone refill and reporting overdose reversal. Chi-2 test was used for variable comparison between groups. Factors associated with overdose reversals were examined by logistic regression analysis. Reasons for naloxone refill, overdose situation and management were presented descriptively.

**Results:**

Among 1,079 study participants, 22% (*n* = 235) returned for naloxone refill, of which 60% (*n* = 140) reported a total of 229 overdose reversals. Reversals were more likely to be reported by participants trained at needle exchange programs (NEPs) [adjusted odds ratio (AOR) = 5.18, 95% Confidence interval (CI) = 3.38–7.95)], with previous experience of own (AOR = 1.63, 95% CI = 1.03–2.58) or witnessed (AOR = 2.12, 95% CI = 1.05–4.29) overdose, or who had used sedatives during the last 30 days before initial training (AOR = 1.56, 95% CI = 1.04–2.33). A majority of overdoses reportedly occurred in private settings (62%), where the victim was a friend (35%) or acquaintance (31%) of the rescuer.

**Conclusion:**

Participants with own risk factors associated with overdose (e.g., injection use, concomitant use of benzodiazepines and previous experience of own overdose) were more likely to report administering naloxone for overdose reversal. Overdose management knowledge was high. The findings indicate that implementation of multi-site OPEND reaches individuals at particularly high risk of own overdose in settings with limited previous harm reduction strategies and favors a further scaling up of naloxone programs in similar settings.

## Introduction

Globally, drug related deaths (DRDs) have increased during the last decades (1), where opioids, used alone or together with other drugs, are present in a majority of the cases (2), and is the single most common cause of death among individuals with opioid use disorder (OUD) (3). Although DRDs in Sweden has decreased slightly during 2018 and 2019, Sweden has the highest number (81.5) of DRDs/million in EU (22.3), including UK, Turkey and Norway (2). Key strategies for reducing the harms of OUD include increasing availability and access to NEPs (4, 5), opioid substitution treatment (OST) (6–10), drug consumption rooms (5, 11, 12), and overdose prevention education and naloxone distribution (OPEND) (3, 13–17). Since the mid 1990's naloxone use has gradually changed from medical professionals reversing opioid overdoses in acute medical settings to being a part of harm reduction interventions including training and distributing naloxone for the use of laypersons. Naloxone is a mu-specific opioid antagonist which temporarily reverses respiratory depression caused by opioid overdose. Naloxone has no misuse potential and although adverse events are rare, individuals with a physical opioid dependence may experience distressing withdrawal symptoms, such as vomiting, nausea or agitation (17). Engagement in OPEND among people who use opioids (PWOU), their family members, and community workers is strong and trained bystanders have shown similar skills in overdose intervention as medical experts (18). Overdoses commonly occur in the company of others (19–22) and broad scale access to naloxone and training is essential as it increases the opportunity of safe and efficient opioid overdose reversals, when and where they occur. Research on broad scale OPEND in Massachusetts, USA showed a reduced mortality on a community level with significant reduction in mortality in communities where distribution exceeded 100 enrollments per 100.000 population (16). A systematic review conducted in 2016, by McDonald and Strang, concluded that take-home naloxone (THN) programs are safe and reduce overdose mortality not only among its participants but also on a community level (17).

In many cases, naloxone programs have been introduced in the context of other harm reduction instruments, in settings where such interventions have been a natural development in reducing harm within vulnerable populations (23). Sweden however has historically had a tradition of limited harm reduction services, although OST and NEP were introduced and established early on. Access and availability of these services have traditionally been restricted. Even though a gradual expansion has occurred during the last decade (24, 25), the process has been slow and naloxone distribution is still unequal and insufficient on a national level (26). Therefore, studying the feasibility of broad scale THN implementation and use in a country with an inherent history of zero-tolerance drug policies and control by repression, which may affect acceptability, access, and availability, is important for countries in similar situations.

Research has shown that previous experiences of own overdose, or being witness to someone else's overdose, is frequently reported among OPEND participants (27, 28). PWUO themselves are more likely to report naloxone administration while responding to a suspected opioid overdose (henceforth referred to as an overdose reversal), compared to other groups (29, 30). PWUO at risk of own overdose have shown to be more involved in networks of individuals with similar lifestyles and are thus more likely to witness and act upon overdoses (16, 29, 31). In addition, previous experience of having witnessed overdose(s), having used heroin (29, 31) or methamphetamine (31), have been found to be associated with naloxone refill and reports of reversals, suggesting that the primary OPEND focus should be to reach at-risk populations (29, 30). Overdose management, and especially seeking emergency medical assistance (EMA), has shown to vary in different settings and populations. A systematic review, including mainly OPEND programmes based in USA, found fear of police involvement to be the most common self-reported reason for not seeking help (28).

This study aims to describe participant characteristics and to identify factors associated with returning for naloxone refills and having used naloxone for overdose reversal. Additionally, this study aims to examine reasons for naloxone refills, and to describe the self-reported overdose situation and management thereof. To the best of our knowledge, this has not previously been done in a large-scale OPEND program exclusively distributing highly concentrated intra-nasal (IN) naloxone. Given the previously low implementation of differentiated harm reduction programs in the present setting, it is of relevance to address the opportunity of OPEND to reach large numbers of at-risk individuals through an already existing extensive network of public health sites.

## Materials and Methods

### Setting

This study was conducted in Skåne county, southern Sweden, with a population of ~1.36 million inhabitants (32). Swedish healthcare, including OST, public and private, is tax-financed and covered by the Swedish universal health insurance, which makes healthcare strongly subsidized. Swedish counties are self-governing which includes responsibilities for healthcare provision, both private and public. This has led to national differences concerning provision of healthcare for individuals suffering from OUD, where access to and availability of both OST and NEP is essentially greater in Skåne county, compared to other counties.

All four NEPs are integrated parts of the Infectious Disease Departments in the county and are regulated by the syringe and needles exchange act (33, 34). Their staff consists of physicians and nurses specializing in infectious diseases, experienced in managing medical emergencies, providing visitors with an array of services besides distributing enough injection equipment to make sure every injection occurs with new and sterile equipment. All costs for services and injection equipment provided by NEPs are tax-financed and free of charge. After the increased access to OST in the county, many current NEP participants report injecting stimulants. However, mixed use of opioids and stimulants is prevalent. Dynamic interaction occurs between OST and NEPs, with patients participating in both intermittently.

OST is provided by specialist healthcare and requires OUD for at least 1 year, and a minimum of 20 years of age, with the possibility of making acceptations to the latter recommendation (35). In addition to pharmacological treatment with methadone or buprenorphine, OSTs are required to provide psychosocial/psychological treatment, basic somatic healthcare, and regular testing for blood-borne infections.

Naloxone can only be prescribed to laypersons at risk of overdose, given that the prescribing physician has provided information on overdose recognition and overdose management (36). As of November 2018, naloxone can also be prescribed by registered nurses (37). Prevention education and training and THN was offered free of charge to patients visiting any of the included sites, regardless of patient's interest of participating in the study. The naloxone kit contained two doses of highly concentrated IN naloxone spray (1.8 mg/ml), vinyl gloves, breathing mask, wipes, “easy-to-use” instructions, a certificate stating participation in THN training session and a card informing potential overdose victim that they had received naloxone due to having suffered from an opioid overdose.

### Study Participants

Recruitment of study participants began in conjunction with OPEND implementation in June 2018 and continued until December 2020. OPEND was implemented at 31 sites, including all NEPs (*n* = 4), all OST programs (*n* = 22) in the county. Included were also in-patient addiction treatment facilities (*n* = 3) and two outpatient addiction unit mainly servicing patients not enrolled in OST programs. As of December 2020, the number of patients attaining overdose prevention education and training and had received an initial naloxone kit had reached 1,700, of which data from 1,079 individuals were eligible for study inclusion. Among those who received training and THN 524 declined taking part in the study. Written consent was missing for 69 of the individuals who had filled out the questionnaire(-s) and had to be ruled out, whereas 28 individuals had filled out the initial form twice. A written informed consent was signed by participants after receiving written and oral information about the study. No economic compensation was provided for study participation. The study was conducted in accordance with the Declaration of Helsinki 2013 (38) and was approved by the Regional Ethics Board, Lund (file no. 2018/300).

In accordance with applicable regulations naloxone may only be prescribed to individuals at risk for opioid overdose. Training curriculum was also provided to professional partners in the target group network, encouraging them to ask their clients if they had a kit, and if so, where it was kept. These individuals are not included in this material.

### Opioid Overdose Prevention Training

The OPEND training curriculum previously described in detail in Troberg et al. (39) is based on a train-the-trainer model where the project leaders train staff at all sites, whom in turn train their patients. The train-the-trainer education sessions encourages trainers to include patients on a broader level. Patients are also given information material to pass on to others and are also encouraged to inform others on how to identify an opioid overdose, what to do in case of witnessing an overdose, and where their naloxone is kept.

Patients could turn to either NEP or OST (wherever enrolled), for naloxone refill regardless of initial training site. If initial kit and training had been provided at an in-patient site the patients were recommended to turn to the non-OST out-patient site for refill.

The train-the-trainer model aim for each site to be self-sufficient when it comes to training new coworkers. However, the project leaders continuously offer support and training for staff at newly established sites, or to avoid high turnover leading to discontinuation in OPEND delivery, the project leaders are responsible for support and re-training at these sites.

Training and distribution continued during the Covid-19 pandemic, though patients' practical (hands on) practice on the CPR-manikin was paused, in accordance with government recommendations and restrictions. Train-the-trainer sessions (39) were held outdoors, or on-line, to accommodate new sites opening during the pandemic. Exceptions were only made if deemed necessary, and if requirements on safety precautions could be met.

Key trainers from each unit were invited to a naloxone conference twice a year, to enhance networking and information sharing on research developments in the field, project developments, facilitators, challenges, and results. During the COVID-19 pandemic meetings were held on-line.

### Data Collection

Upon completion of training, patients were informed about the study and offered participation. An informed consent was obtained, and patients were asked to fill out the questionnaire. A full description of the questionnaires has previously been described in Troberg et al. (39). The following description of collected data only concerns questions currently applied in this study.

All included sites gradually replaced paper questionnaires with digital ones from November 2018. Clinical Trials Skåne was responsible for creating the digital solution of the questionnaire and to store collected data. The project provided all sites with iPads with the pre-installed Research Electronic Data Capture (REDCap) application, for collecting and storing the information. Upon patients request support on filling out the questionnaire was provided by employees at each unit. Paper questionnaires, used in the beginning of the study, were manually entered into the RedCap database by a research secretary, with no other involvement in the project.

The initial questionnaire included data on demographics, substance use during last 30 days, lifetime experiences of own or witnessed overdose(s) and on how to recognize and respond if witnessing an overdose. Follow-up data upon naloxone refill included questions on what had happened to the previous naloxone/kit (used on self/others, lost, stolen or other/free-text option). If naloxone was reported to have been used for overdose reversal, collected data concerned victims' overdose symptoms (irregular/no breathing, blue lips/fingertips, pale/cold/weak pulse, unconscious), number of naloxone dose(s) administered and to whom (man, woman, other and relative, friend, acquaintance, stranger, other), applying complementary overdose prevention strategies (calling ambulance, rescue breathing, recovery position, other/free-text option), and where the overdose had been taken place (public setting, private own accommodation, someone else's private accommodation, other). If the respondent answered that ambulance had been called, they were asked if they stayed with the overdose victim until ambulance arrival.

### Data Management

Quality control of RedCap data was executed by K.T. and P.I. during February and March 2021. If baseline data had been collected on more than one occasion, only data from the first training session was included, which resulted in exclusion of 28 cases. Social security numbers which were not entered correctly were traced and corrected. All paper questionnaires were matched to the written consent documents. Data had to be excluded for 69 individuals since consent was confirmed to be missing.

As data from out-patient addiction care facilities included a limited number of study participants (*n* = 13) their data was merged with data provided by OST-sites, also providing out-patient addiction care.

Six variables were recoded. Substance use during previous 30 days to inclusion were recoded to “opioids” if the respondent had replied “heroin,” “fentanyl,” “methadone,” “buprenorphine,” or free text answers classed as opioids (i.e., morphine, Tramadol, and oxycodone). If the respondent had replied “benzodiazepines,” “zopiclone,” “zolpidem,” “pregabalin” or sedatives described in the free text variable [i.e., “barbiturates” and “gamma-hydroxybutyrate (GHB)”], these variables were recoded to “sedatives.” “Cocaine” or any stimulants described in the free text variable (i.e., “amphetamine,” “methamphetamine,” “MDMA” and pharmaceuticals used for treating attention deficit/hyperactivity disorder) were recoded to “stimulants.” Hallucinogenic substances such as “LSD” and “psilocybin,” substances such as “cannabis,” “spice,” or substances noted as “other” were recoded into to “other.” “Heroin,” “fentanyl,” or use of OST-medication (“methadone,” “buprenorphine” or “Suboxone”) among participants not trained in an OST facility, or participants reporting opioid substances in free text (ibid) were recoded to “illegal opioids.” Also, apart from administrating naloxone, additional recommended responses to overdose management, such as “called ambulance,” “rescue breathing,” “recovery position,” were recoded to “measures taken when witnessing an overdose (most recent).”

If any of the options “yes, it was used to reverse opioid overdose on myself” or “…. to reverse opioid overdose on someone else” was selected, followed by a clear statement in free text where the participant denied having used their naloxone, corrections were made. This led to changes (a decrease) in numbers of reversals reported in 11 cases. One participant first reported not having used naloxone, followed by a free text explanation clearly stating having used naloxone to reverse overdose and on whom, led to inclusion this data as “yes, it was used to reverse opioid overdose on someone else,” increasing numbers of reversals by one.

Answering “other use” on “Drug use during last 30 days” followed by a substance in free text which was of no interest in regard to this study, such as medication for asthma or high blood pressure, led to ruling out “other use” in 16 cases.

When respondents had replied a range of numbers, the mean number (rounded to the lower integer) was used. Variables describing numbers of previous experiences of own or witnessed overdose, were recoded as “1 OD experience,” “2-4 OD experiences” and “5 or more OD experiences.”

### Data Analysis

Descriptive statistics was used to examine demographic and behavioral characteristics of the study sample, of those returning for naloxone refill and of participants reporting overdose reversal. Chi-square test was employed comparing participants returning to those not returning for naloxone refill and for comparing participants reporting reversals, in relation to those returning who did not. Descriptive statistics were also used examining distribution of initial doses, reasons for naloxone refill and the overdose situations in which naloxone had been used to reverse overdose on someone else.

**Figure 1:**
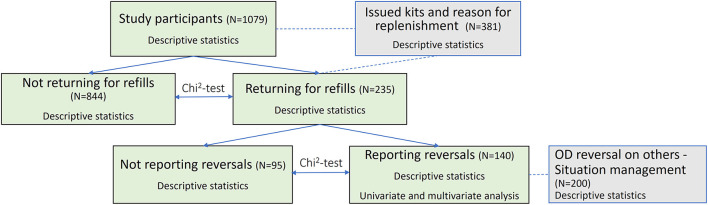
Schematic overview of the data analysis.

Univariate and multivariate logistic regression models were used to identify factors associated with reporting the use of naloxone to reverse opioid overdose, in a sub-sample of individuals returning for naloxone refill. We included age, gender, and variables with a previously documented association with opioid overdose (NEP participation, prior experience of OD, prior witnessing of OD, and use of sedatives) in the analyses. NEP participation was a proxy variable for active drug use (for a schematic overview of the analysis, see Figure 1). Variable correlation of <0.7 was accepted (Supplementary Table A).

SPSS version 27.0 was used for statistical analysis (40).

## Results

### Characteristics of the Study Sample

The majority of study participants were male (68%), received training and initial THN-kit at one of the OST clinics (74%), while 15 and 11% were patients recruited at NEPs and in-patient treatment facilities, respectively. Age varied between 18 and 74 years, with an average age of 40.2 years [standard deviation (SD) = 11.2]. Two thirds reported previous experience of own opioid overdose, of whom more than three quarters reported multiple overdose experiences. Nearly one third reported five or more previous overdose experiences. Having witnessed someone else overdose on opioids was reported by 81%. Nearly all stated that they would know what to do in case of witnessing future overdose and having the intention of calling an ambulance (Table 1).

**Table 1:** Baseline demographic and behavioral characteristics of naloxone study participants (*N* = 1,079).

	**All participants % (*n*)**
**Training and initial kit**	
Needle exchange programs	15.3 (165/1,079)
Opioid substitution treatmenta	73.5 (793/1,079)
In-patient facilities	11.2 (121/1,079)
**Gender**	
Male	68.3 (737/1,079)
Female	31.7 (342/1,079)
**Age**	
Mean (SD) age (years)	40.2 (11.2)
Median age (range)	39 years (18–74)
18–29 years	19.7 (213/1,079)
30–39 years	33.3 (359/1,079)
40–49 years	24.9 (269/1,079)
>50 years	22.1 (238/1,079)
**Lifetime own overdose experience**	
Yes	61.4 (610/993)
1 overdose experience	23.4 (131/560)
2-4 overdose experiences	45.5 (255/560)
5 or more overdose experiences	31.1 (174/560)
No	38.6 (383/993)
**Lifetime experience of witnessing overdose**	
Yes	81.1 (810/999)
Witnessed 1 overdose	17.7 (125/705)
Witnessed 2–4 overdoses	41.6 (293/705)
Witnessed 5 or more overdoses	40.7 (287/705)
No	18.9 (189/999)
**Know what to do when witnessing an overdose**	
Yes	96.7 (982/1,015)
Giving naloxone	94.7 (930/982)
Calling an ambulance	96.4 (947/982)
Performing rescue breathing	88.5 (869/982)
Placing person in recovery position	86.3 (847/992)
Staying until ambulance arrival	87.1 (855/992)
No	1.7 (17/1,015)
Other	1.6 (16/1,015)
**Substance use previous 30 days** ^b^	
Any substance use (including alcohol)	88.7 (922/1,040)
Opioids	77.4 (805/1,040)
Illegal opioids	25.8 (268/1,040)
Sedatives	42.6 (443/1,040)
Alcohol	18.8 (195/1,040)
Stimulants	16.4 (170/1,040)
Other	7.2 (75/1,040)

*Due to missing information, denominators are smaller in numbers in relation to total number of participants*.

*^a^Outpatient addiction treatment facilities included*.

b *Among those stating that they explicitly not wanted to answer the question (n = 27), the majority were trained at OSTs (n = 21), while a minority were trained at NEPs (n = 3), in-patient sites (n = 2) and non-OST out-patient care (n = 1)*.

Three quarters reported to have consumed opioids during the previous 30 days, of which one quarter was noted as illegal opioids. Sedatives were reported by 42%, one in five reported alcohol use, while use of stimulants were slightly less reported (Table 1).

### Characteristics of Participants Returning for Naloxone Refill

In relation to participants not returning for naloxone refill, those who did return were more likely to have received training and initial naloxone kit through NEP and more frequently reported previous experience of own, and witnessed, overdose. Illegal opioid use, as well as use of sedatives, stimulants, and alcohol were more frequently reported by participants returning for naloxone refill (Table 2).

**Table 2:** Baseline characteristics of participants returning for naloxone refill and for those who did not (*N* = 1,079).

	**Returned for refill –all causes % (*n* = 235)**	**Not returning for refill % (*n* = 844)**	**Pearson Chi^**2**^ *p*-value**
**Training and initial kit**			
Needle exchange programs	34.3 (81/235)	10.0 (84/844)	<0.01^*^
Opioid substitution treatment^a^	57.4 (135/235)	78.0 (658/844)	<0.01^*^
In-patient facilities	8.1 (19/235)	12.1 (102/844)	0.09
**Gender**			
Male	71.1 (167/235)	67.5 (570/844)	0.30
Female	28.9 (68/235)	32.5 (274/844)	
**Age**			0.33
Mean (SD) age (years)	40.4 (10.6)	40.1 (11.4)	
Median age (range)	38 (52–73)	39 (56–74)	
**Lifetime own overdose experience**			
Yes	74.3 (150/202)	61.4 (460/749)	<0.01^*^
No	31.7 (64/202)	42.6 (319/749)	
**Lifetime experience of witnessing overdose**			
Yes	89.6 (199/222)	78.6 (611/777)	<0.01^*^
No	10.4 (23/222)	21.4 (166/777)	
**Substance use previous 30 days**			
Any substance use (including alcohol)	88.3 (203/230)	88.3 (719/814)	0.65
Opioids	72.2 (166/230)	78.5 (639/814)	0.11
Illegal opioids	35.7 (82/230)	22.9 (186/814)	<0.01^*^
Sedatives	56.1 (129/230)	38.6 (314/814)	<0.01^*^
Alcohol	26.5 (61/230)	16.5 (134/814)	<0.01^*^
Stimulants	23.0 (53/230)	14.4 (117/814	<0.01^*^
Other	7.8 (18/230)	7.0 (57/814)	0.66

* *p ≤ 0.05. Due to missing information, denominators are smaller in numbers in relation to total number of participants*.

a *Outpatient addiction treatment facilities included*.

### Characteristics of and Factors Associated With Participants Reporting Overdose Reversal

In a sub-sample of individuals returning for naloxone refill participants who had received training and initial kit at NEP were more likely to report naloxone having been used for reversal, while participants trained at OST more often reported other causes upon return (Table 3).

**Table 3:** Baseline characteristics of participants reporting overdose reversal and those who did not (*n* = 235).

	**Returning—reported reversal % (*n* = 140)**	**Returning—other causes for refill ^a^ % (*n* = 95)**	**Pearson Chi^b^ *p*-value**
**Training and initial kit**			
Needle exchange programs	40.7 (57/140)	25.3 (24/95)	0.01^*^
Opioid substitution treatment^a^	50.7 (71/140)	67.4 (64/95)	0.01^*^
In-patient facilities	8.6 (12/140)	7.4 (7/95)	0.74
**Gender**			0.66
Male	70.0 (98/140)	72.6 (69/95)	
Female	30.0 (42/140)	27.4 (26/95)	
**Age**			0.44
Mean (SD) age (years)	41.1 (10.3)	39.4 (11.1)	
Median age (range)	39 (52–73)	37 (45–66)	
**Lifetime own overdose experience**			0.38
Yes	72.3 (94/130)	66.7 (56/84)	
No	27.7 (36/130)	33.3 (28/84)	
**Lifetime experience of witnessing overdose**			0.08
Yes	92.5 (124/134)	85.2 (75/88)	
No	7.5 (10/134)	14.8 (13/88)	
**Substance use previous 30 days**			
Any substance use (including alcohol)	88.3 (121/137)	88.2 (82/93)	0.98
Opioids	72.3 (99/137)	72.0 (67/93)	0.98
Illegal opioids	39.4 (54/137)	30.1 (28/93)	0.15
Sedatives	57.7(79/137)	53.8 (50/93)	0.57
Alcohol	25.6 (35/137)	28.0 (26/93)	0.68
Stimulants	26.3 (36/137)	18.3 (17/93)	0.16
Other	7.3 (10/137)	8.6 (8/93)	0.72

*Due to missing information, denominators are smaller in numbers in relation to total number of participants*.

* *p ≤ 0.05*.

a *Other reason = lost, stolen, expired, other reason or not known*.

b * Outpatient addiction treatment facilities included*.

Logistic regression analysis showed that initial training provided by NEP, previous experience of own overdose, being witness to someone else's overdose, and use of sedatives during the last 30 days prior to inclusion, were associated with having reported overdose reversal (Table 4).

**Table 4:** Baseline data associated with use of naloxone for overdose reversals (*n* = 235).

	**Univariate analysis OR (95% CI)**	***P*-value**	**Multivariate analysis AOR (95% CI)**	***P*-value**
Male gender	1.09 (0.74–1.61)	0.64	1.04 (0.68–1.59)	0.86
Age in years	1.01 (0.99–1.02)	0.29	1.02 (1.00–1.03)	0.09
Initial training at needle exchange program	5.28 (3.57–7.82)	<0.01^*^	5.18 (3.38–7.95)	<0.01^*^
Prior experience of own overdose	1.76 (1.17–2.64)	<0.01^*^	1.63 (1.03–2.58)	0.04^*^
Prior experience of witnessing overdose	3.24 (1.66–6.29)	<0.01^*^	2.12 (1.05–4.29)	0.04^*^
Use of sedatives previous 30 days	2.41 (1.43–2.93)	<0.01^*^	1.56 (1.04–2.33)	0.03^*^

* *p ≤ 0.05*.

### Reason for Naloxone Refill

Naloxone refill was requested on 381 occasions by 235 unique individuals. Even though 73% of initial kits and training were provided by OST clinics, a near equal number of naloxone refills were rendered at NEPs and OSTs (176 and 175, respectively), whereas a minor number were replenished at in-patient facilities. NEP reported the highest numbers of reversals in relation to trained individuals (75%). Subsequently, mean numbers of naloxone refills were highest at the NEPs (2.2) and lowest at the OSTs (1.6). Percentages of reports referring to THN-kit being lost were quite similar in all types of sites, however, previous kit “given to someone else” was more frequently reported as a reason for refill at the OSTs (18 vs. 10%). A similar pattern was noted regarding not knowing what had happened to the previous kit, where the highest percentage was reported at OSTs compared both to NEPs and at in-patient facilities (Table 5).

**Table 5:** Naloxone refills and reason for refill (*n* = 381).

	**All sites**	**Needle exchange programs**	**Opioid substitution treatment^a^**	**In-patient facilities**
Refills (*n*)	381	176	175	30
Individuals reporting refill (*n*)	235	81	135	19
Mean number of refills (SD)	1.6 (1.16)	2.2 (1.64)	1.3 (0.56)	1.6 (0.96)
Median number of refills (range)	1 (8;1–9)	2 (8;1–9)	1 (2;1–3)	1 (3;1–4)
Per trained individual [% (*n*)]	35.3 (381/1,079)	106.7 (176/165)	22.1 (175/793)	24.8 (30/121)
Reports of >3 refills (*n*)^b^	11	10	0	1
**Reasons for refill**				
Overdose reversals [% (*n*)]	60.1 (229/381)	69.9 (123/176)	50.9 (89/175)	56.7 (17/30)
On other [% (*n*)]	52.5 (200/381)	61.9 (109/176)	44.6 (78/175)	43.3 (13/30)
On self [% (*n*)]	7.6 (29/381)	8.0 (14/176)	6.3 (11/175)	13.3 (4/30)
Per trained individual [% (*n*)]	21.2 (229/1,079)	74.5 (123/165)	11.2 (89/793)	14.0 (17/121)
Per distributed kit [% (*n*)]	15.7 (229/14,160)	36.1 (123/341)	9.2 (89/968)	11.3 (17/151)
Lost [% (*n*)]	17.6 (67/381)	17.6 (31/176)	17.1 (30/175)	20.0 (6/30)
Given to someone else [% (*n*)]	13.4 (51/381)	9.7 (17/176)	17.7 (31/175)	10.0 (3/30)
Other [% (*n*)]	2.9 (11/381)^c^	1.7 (3/176)	2.9 (5/175)	10.0 (3/30)
Not known [% (*n*)]	6.0 (23/381)	1.1 (2/176)	11.4 (20/175)	3.3 (1/30)

a *Outpatient addiction treatment facilities included (n = 2)*.

b *These participants reported 16% (n = 60) of all refills. Nine of them reported reversing overdoses on others, resulting in 21% (n = 41) of all overdose reversals*.

c *Stolen n = 7; Police confiscated n = 1; Expired n = 3*.

Eleven participants returned for naloxone refill on more than three occasions, representing 16% (*n* = 60) of all refills. Compared to participants reporting reversals, this group contained a larger proportion of women (45%). All but one participant had received training and initial naloxone kit by NEP (Table 5).

### Characteristics of the Overdose Situation

In cases where naloxone had reportedly been used on someone else (*n* = 200) a majority reported that the overdose had occurred in a private accommodation (62%), that the overdose victim was male (70%), and that the victim was a friend or an acquaintance (65%). In 15%, respectively, 20% of the cases the victim was reported to be a relative or a stranger. Apart from administering naloxone, additional overdose management activity was performed by 74%. Nearly half of the respondents (46%) reported calling an ambulance, while rescue breathing and placing the victim in recovery position was applied in 38 and 43%, respectively. One or two doses of naloxone were administered in 48% of the cases, respectively, while a minority reported administering more than two doses (Table 6).

**Table 6:** Situation where naloxone was reported to have been used to reverse someone else's overdose (*N* = 200).

	**% (*n*)**
**Most recent overdose took place in…**	
Own accommodation^a^	29.1 (55/189)
Someone else's accommodation a	32.8 (62/189)
Public place^b^	34.9 (66/189)
Other	3.2 (6/189)
Overdose symptoms^*^	
Irregular/no breathing	54.4 (105/193)
Blue lips/fingertips	67.9 (131/193)
Pale/cold/weak pulse	43.5 (84/193)
Unconscious	65.3 (126/193)
**Naloxone was used on**	
A man	70.4 (138/196)
A woman	27.6 (54/196)
Other	1.0 (2/196)
**Relationship to the victim**	
A relative	15.3 (28/194)
A friend	34.7 (68/194)
An acquaintance	30.6 (60/194)
Stranger	17.5 (34/194)
Other	2.1 (4/194)
Measures taken when witnessing an OD (most recent)^*^	74 (148/193)
Called ambulance	45.9 (89/194)
Stayed with person until ambulance arrived	76.4 (68/89)
Rescue breathing	38.7 (75/194)
Recovery position	43.3 (84/194)
Other^c^	12.4 (24/194)
**Number of doses used**	
One	47.8 (87/182)
Two	47.8 (87/182)
Three or four	4.4 (8/182)
Reason for not calling ambulance^*^	
Afraid police would turn up	14.0 (14/100)
Did not think it was necessary	60.0 (60/100)
The victim did not want me to	23.0 (23/100)
Fear of social services getting involved	4.0 (4/100)
Other^d^	17.0 (17/100)

*Due to missing information, denominators are smaller in numbers in relation to total number of participants*.

* *Multiple answers possible*.

a *Ambulance was called in 39.3% of the cases when overdose occurred at a private accommodation*.

b *Ambulance was called in 53.0% of the cases when overdose occurred in a public place*.

c *Other (free text): stayed with person (n = 10), Asked someone else to stay (n = 2), CPR (n = 2), Someone else intervened (n = 1), Shower/cold water (n = 3), Pain stimulation (n = 1)*.

d *Other: Someone else called (n = 4), No phone (n = 1), Became scared (n = 1), Did not want to get involved (n = 1); Paramedics at scene already (n = 1), Person already dead (n = 1)*.

A majority of respondents did not find it necessary to call for ambulance (60%) or refrained from calling due to the victim not wanting responder to call (20%). Fear of police or social services involvement was reported as reason for not seeking EMA by 14 and 4%, respectively. One or two doses of naloxone were administered in 48% of the cases, respectively, while a minority reported administering more than two doses (Table 6).

## Discussion

The results from this study show that a large proportion of participants returned for naloxone refill and reported naloxone administration for overdose reversal implying that this naloxone project did reach a proportion of at-risk individuals efficiently. Our findings show self-reported overdose reversal to be associated with having received training and initial kit through NEPs, previous experience of own and witnessed overdose, and recent use of sedatives. This correlates partially with findings where previous experience of witnessing overdose (31) is associated with overdose reversal. Over one fifth of our study participants returned for naloxone refill, of which the majority (60%) reported having used previous naloxone kit for overdose reversal. International comparisons of these results can be difficult due to differences in regulations and setting. Reports on refills in relation to participants is not a commonly presented measure, however Enteen et al. (41) found that 24% returned for refill, which is similar to our result. Proportion of naloxone having been used for overdose reversal, in relation to all cause refills, varied between 39-69% (41–45).

A majority reported that the overdose had occurred in a private accommodation, that the victim had been male and was a friend or an acquaintance of the rescuer, which to a large extent correlates with other studies (16, 31, 43, 46, 47).

With few exceptions, international studies on OPEND generally include more male participates (60–70%) between 35–40 years of age (28, 31, 44) which was also true for our participants. The majority of study participants had received training and initial naloxone kit through OSTs, mirroring the high accessibility and availability of OST in the county. A large proportion of study participants reported previous experience of own overdose and, to an even higher extent, of having previous experience of witnessing someone else's overdose, which is concordant to international findings concerning OPEND participants with previous or current opioid use (28). Minor differences between participants trained at NEPs, OSTs and in-treatment facilities were found, where trainees at NEPs consisted of a higher percentage of women, while in-patient trainees were younger than those trained at NEPs or OSTs. Percentage of lifetime experience of own overdose was slightly higher among in-patient trainees, while having witnessed overdose was highest among NEP trainees (Supplementary Table B). Similar to research by Rowe et al. (31), our result show OST-enrollees to be less likely returning for naloxone refill and to report having administered naloxone overdose reversal.

Although only a small group returned for naloxone refills on more than three occasions they stood for a considerable proportion of refills and reversals. Compared to participants reporting reversals, this group contained a larger proportion of women, were slightly older and were trained at NEPs. These findings call for further research on overdose situations, settings, and management, to ensure access and availability to appropriate support and the need for continuous training enabling continuant engagement and appropriate overdose management. This group is of importance since they may possess accumulated knowledge and experience (48), however have also shown to report ineffective or counterproductive ways of managing overdose situations (22).

In congruence with previous research (49, 50), participants reporting overdose reversals occurring in a private setting were more reluctant to seek EMA, as opposed to those reporting overdoses occurring in a public setting. Although the numbers in this study were small, participants reporting refraining from seeking EMA due to fear of police or social services involvement were more frequently reported when the overdose occurred in a private setting (Supplementary Table C). This could partly explain why the percentage of respondents who sought EMA were more frequently reported by those reversing overdoses in a public setting. While current study participants generally reported a high level of confidence in overdose management, results show that only 46% sought EMA. A high level of intent (44, 51), vs. a lower rate of actually seeking help when witnessing overdose have been reported in previous studies (28, 42, 51, 52). Concerns on whether THN availability would lead to a decrease in help seeking have been raised. Research on EMA seeking conducted before availability of THN does however show similar response rates of seeking help when dealing with overdose (46), and research on a national naloxone program Scotland showed no evidence of broad scale THN implementation leading to a decrease in ambulance attendance (53).

Reasons for refraining from help seeking varies between settings and groups of individuals over time, however, structural vulnerabilities, such as homelessness or insecure housing arrangements, may impair possibilities to follow policy recommendations, or even stand in direct conflict thereof (54). Fear of police involvement have previously been shown to be the most common reason for not calling EMA (28, 42, 44, 55, 56). Interestingly, this was not the case with our respondents as refraining from calling due to fear of police involvement was relatively low among our participants, whereas the majority stated not finding it necessary, along with one in five reporting that the overdose victim did not want the respondent to call. Similar findings have been reported previously (42, 57), however not to the same extent. Even though overdose situations are complex and do not always allow for management according to protocol (54, 58), three-quarters of participants in our study reported using additional prevention strategies, besides naloxone administration, which is consistent to previous research (41), or low (44) compared to other. Further investigation into decisions concerning overdose management is needed.

Participants reporting other causes for refill than having used previous kit to reverse overdose were more prone to be OST-patients, there were no other significant differences between the two groups. There is a need for further investigation to this group, as it may contain individuals that are highly involved in reversals but for different reasons are not willingly to disclose this information.

This project had the advantages of being politically and financially supported and of having a regional infrastructure allowing for efficient implementation and access to patients at risk through a network of multiple local healthcare sites by utilization of train-the-trainer model (39). As Swedish regulations state that naloxone only can be prescribed to patients at risk of opioid overdose, the goal was to increase awareness and to motivate all patients to accept training and THN offered at targeted sites. During the first 30 months the naloxone program had been implemented at 31 sites providing 1,700 at-risk individuals with training and initial naloxone kit, of which 1,079 (64%) were included in this study. Not included in this study were the extensive collaborations with, and of training staff, not only within the healthcare system but also members of different organizations within the community, including social workers, low threshold housing staff, watchmen, peer- and interest groups working in environments where overdoses occur. Although regulations restrict prescription of naloxone to (members of) these groups, it is still important to increase knowledge and engage individuals on a broader level as it also provides an opportunity to decrease stigma.

For naloxone to be present whenever and wherever overdoses occur, increasing access is vital. Only allowing individuals with a risk of own future overdose to be prescribed naloxone may be insufficient and may also present a risk of further polarization and stigmatization as this clearly separates “us and them.” It also hinders illegal immigrants, individuals wanting to “stay under the radar,” and those working in risk environments from obtaining naloxone, which makes them dependent on knowing who to turn to in case of witnessing an overdose. For Sweden to fulfill WHO guidelines, proposing naloxone to be made available to everyone at risk of witnessing overdose (14), further legal changes are called for. Changes would hopefully pave the way for implementation of more creative and effective ways of reaching those needing it most, such as peer-to-peer education and administration. Although availability of naloxone in Sweden has increased, uneven distribution on a national level due to inequalities in financial capacity does indicate a need for governmental support for equal access to naloxone.

### Limitations

This study relies on retrospective self-reported events, with the risk of participants being subject to social desirability and/or recall bias. Inclusion into the study was optional, meaning participants and those not willing to partake did get the same education and the same access to naloxone and support. This may have contributed to skewness in representation as to patients not having time to respond to questions or not being able to because of abstinence, or even not being able to sign an informed consent. Instead, quick training and THN was prioritized. Although participants were recommended to return for refill as soon as their naloxone had been used/stolen/lost or given to someone else, some may have refrained from returning for refill, or from reporting. Participants also reported giving their kit to someone else in need while returning for refill. It is unlikely that those “in need” would report if they used the naloxone. Depending only on self-reported data could also be a limitation as there were individuals not staying at the scene after administering naloxone until ambulance arrival, and in some cases, ambulance took the victim to the emergency unit, leaving the rescuer not knowing if the victim survived or not.

Stigma and perceived risk of being punished, especially in OST, on reporting reversal upon naloxone refill, and a social desirability of being a “good” patient may incline patients to prefer reporting previous dose as lost or stolen, rather than being administered for treatment of a suspected opioid overdose on members of family, friends, or even themselves. Anonymous participation in Swedish NEPs is not possible since the syringe and needles exchange act requires registration and identification by social security number. This limited access can be a barrier to obtain THN as well as other harm reduction services. Social security number is mandatory when it comes to all healthcare in Sweden, including NEP and provision of naloxone, since it must be prescribed.

Findings from this study may differ from other regions, as the study was limited to one county in Sweden. In a jurisdictional context, OPEND could only be offered to those themselves at risk of opioid overdose, which may limit comparability with international programmes with inclusion of bystanders in general.

A further potential limitation is the fact that the present study includes the first 9–10 months of the COVID-19 pandemic, an event with potential to present a challenge to many clinical or patient-centered outreach interventions. In the present setting, an active transmission of the virus and virus-preventing restrictions were ongoing since mid-March, 2020. However, as the Swedish policy toward COVID-19 never involves any lock-down or confinement measures during the present period, the naloxone training and distribution could be maintained throughout this period, although with adaptations in the training practices. Thus, while this makes it less probable that the outcome variables in the present study would change substantially during the pandemic, data were reviewed with this regard. However, a full sensitivity analysis of pre-COVID vs. COVID-affected periods would likely not rule out this potential limitation. Time from naloxone training to first refill occasion was on average more than 9 months, such that the COVID-19-affected proportion of the follow-up period was too short for a full assessment of whether COVID-19 affected the principal study measures of the present study; even individuals trained in close temporal association with the pandemic outbreak would have an insufficient follow-up time for any such impact to the reliably demonstrated. Currently, the issue of COVID-19 in naloxone distribution is assessed from the present project in a separate sub-project, and will be analyzed scientifically, but goes beyond the scope of the present work and will be published elsewhere.

### Conclusion

The findings from this study add to the growing body of evidence showing that although requiring individual prescription, sufficient and effective multi-site OPEND can reach and engage at-risk individuals on a broad level. Even though the majority had received training and initial kit through OSTs, overdose reversals were more commonly reported by participants trained at NEPs. Patients reporting own risk factors associated with overdose were more likely to return for refill and of reporting overdose reversal. Self-reported knowledge about overdose management was high. In most cases naloxone was used on someone else than the person which had been prescribed naloxone, indicating a need for law changes making naloxone available to those at risk of witnessing overdose and allowing peer-to-peer education and administration. Naloxone programs in a setting with previously low implementation of harm reduction measures, appear to be feasible, and findings support up-scaling of naloxone programs.

## Data Availability Statement

The SPSS data used to support the findings of this study are restricted by the Regional Ethics Board, Lund, Sweden, in order to protect patient privacy. Data are available from Katja Troberg, katja.troberg@med.lu.se, for researchers who meet the criteria for access to confidential data.

## Ethics Statement

The studies involving human participants were reviewed and approved by Regional Ethics Board, Lund (file no. 2018/300). The patients/participants provided their written informed consent to participate in this study.

## Author Contributions

KT is a Ph.D. student in the project and the principal writer of this manuscript. KT, PI, and MB were responsible for developing information and educational material, with MB contributing with scientific and medical advice. KT and PI were both project leaders and responsible for implementation and management of the naloxone project. In her role as co-supervisor of KT and DD made scientific contributions to the manuscript, which was also provided by AH, supervisor of KT and the principal investigator of the study. All authors contributed to the article and approved the submitted version.

## Funding

This work was financially funded by grants from Southern Health Care Region and Region Skåne (Sweden) to KT, DD, MB, and AH.

## Conflict of Interest

AH holds a position at Lund University sponsored by the Swedish state-owned gambling operator AB Svenska Spel. He disposes research grants from the research councils of AB Svenska Spel, the state-owned alcohol monopoly Systembolaget, the Swedish Enforcement Agency, and the Swedish Sports Federation. He is currently involved in a clinical research study which receives non-financial support from the commercial body Kontigo Care in digital follow-up tools in the treatment of addictive disorders. AH is the national principal investigator of a prior pharmaco-epidemiological survey study conducted by the US research institute Research Triangle Institute and which was sponsored by a pharmaceutical company (Shire), which supported the study but did not pay any personal fees to AH as an individual researcher. The present competing interests are not involved in the present project. The remaining authors declare that the research was conducted in the absence of any commercial or financial relationships that could be construed as a potential conflict of interest.

## Publisher's Note

All claims expressed in this article are solely those of the authors and do not necessarily represent those of their affiliated organizations, or those of the publisher, the editors and the reviewers. Any product that may be evaluated in this article, or claim that may be made by its manufacturer, is not guaranteed or endorsed by the publisher.
