# Supplementary material for: Characteristics of and Experience Among People Who Use Take-Home Naloxone in Skåne County, Sweden
Source: Front Public Health. 2022 Mar 10;10:811001. doi: 10.3389/fpubh.2022.811001 (PMC8960176; doi:10.3389/fpubh.2022.811001)
Supplement: Supplementary file 1 [file Data_Sheet_1.PDF]

Supplementary file

Table a. Correlation analysis

		Gender	Age	Initial training NEP	Prior experience of own OD	Prior experience of witnessing OD	Use of sedatives previous 30 days
Gender	Pearson Correlation	1	,062*	-0,048	0,039	-0,001	-0,015
	Sig. (2-tailed)		0,043	0,114	0,222	0,970	0,634
	N	1079	1079	1079	993	999	1079
Age	Pearson Correlation	,062*	1	0,011	-0,061	0,057	-,062*
	Sig. (2-tailed)	0,043		0,719	0,056	0,072	0,041
	N	1079	1079	1079	993	999	1079
Initial training NEP	Pearson Correlation	-0,048	0,011	1	-0,030	,083**	,164**
	Sig. (2-tailed)	0,114	0,719		0,349	0,008	0,000
	N	1079	1079	1079	993	999	1079
Prior experience of own OD	Pearson Correlation	0,039	-0,061	-0,030	1	,314**	,114**
	Sig. (2-tailed)	0,222	0,056	0,349		0,000	0,000
	N	993	993	993	993	958	993
Prior experience of witnessing OD	Pearson Correlation	-0,001	0,057	,083**	,314**	1	0,047
	Sig. (2-tailed)	0,970	0,072	0,008	0,000		0,135
	N	999	999	999	958	999	999
Use of sedatives previous 30 days	Pearson Correlation	-0,015	-,062*	,164**	,114**	0,047	1
	Sig. (2-tailed)	0,634	0,041	0,000	0,000	0,135	
	N	1079	1079	1079	993	999	1079

*. Correlation is significant at the 0.05 level (2-tailed).

**. Correlation is significant at the 0.01 level (2-tailed).

Table b. Baseline characteristics of naloxone study participants

	NEP (N=165)	OST (N=793) [a]	In-patient (N=121)
**Gender**			
Male	63.0 (104/165)	68.6 (544/793)	73.6 (89/121)
Female	37.0 (61/165)	31.4 (249/793)	26.4 (32/121)
**Age**			
Mean (SD) age (years)	40.4 (11.7)	40.9 (11.0)	35.1 (10.6)
Median age (range)	38 years (55; 18-73)	39 (54;20-74)	33 (43; 18-61)

**Lifetime own OD experience**			
Yes	58.1 (90/155)	61.8 (455/736)	63.7 (65/102)
No	41.9 (65/155)	38.2 (281/736)	36.3 (37/102)
**Lifetime experience of witnessing OD**			
Yes	88.6 (140/158)	80.0 (583/729)	77.7 (87/112)
No	11.4 (18/158)	20.0 (146/729)	22.3 (25/112)
**Substance use previous 30 days** [b]			
Any substance use (including alcohol)	91.5 (151/165)	83.9 (665/793)	87.6 (106/121)
Opioids	61.8 (102/165)	77.3 (613/793)	74.4 (90/121)
Illegal opioids	63.0 (104/165)	11.7 (93/793)	43.8 (53/121)
Sedatives	60.0 (99/165)	31.7 (251/793)	76.9 (93/121)
Alcohol	32.7 (54/165)	12.4 (98/793)	35.5 (43/121)
Stimulants	50.3 (83/165)	6.8 (54/793)	27.5 (33/120)
Other	12.7 (21/165)	5.3 (42/793)	10.7 (13/121)

[a] Outpatient addiction treatment facilities included.

[b] Among those stating that they explicitly not wanted to answer the question (n=27), the majority were trained at OSTs (n=21), while a minority were trained at NEPs (n=3), in-patient units (n=2) and non-OST out-patient care (n=1).

Due to missing information, denominators are smaller in numbers in relation to total number of participants.

Table c. Reason for not calling ambulance - Fear of police or social services involvement (N=14)

Private accommodation	85.7%
Public setting	14.3%
